# Supplementary material for: Rice Husk Cellulose-Based Adsorbent to Extract Rare Metals: Preparing and Properties
Source: Materials (Basel). 2023 Sep 19;16(18):6277. doi: 10.3390/ma16186277 (PMC10533061; doi:10.3390/ma16186277)
Supplement: Supplementary file 1 [file materials-16-06277-s001.zip › materials-2613066-supplementary.pdf]

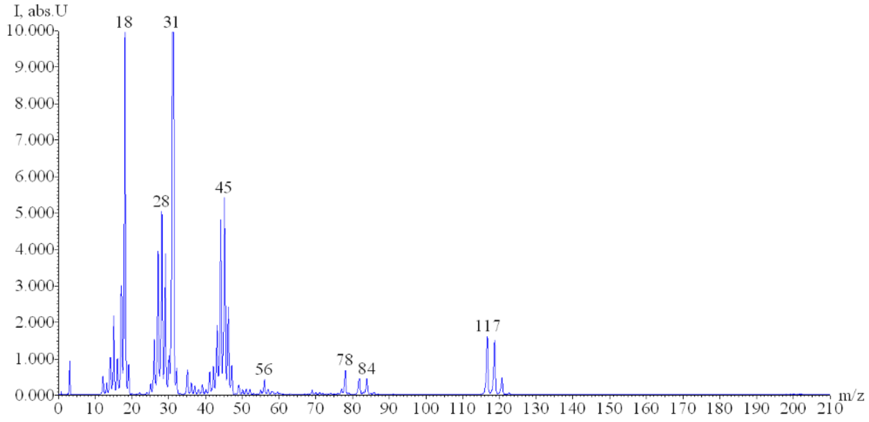
I, abs.U
10.000
9.000
8.000
7.000
6.000
5.000
4.000
3.000
2.000
1.000
0.000
18
31
28
45
56
78
84
117
0 10 20 30 40 50 60 70 80 90 100 110 120 130 140 150 160 170 180 190 200 210
m/z

(a)

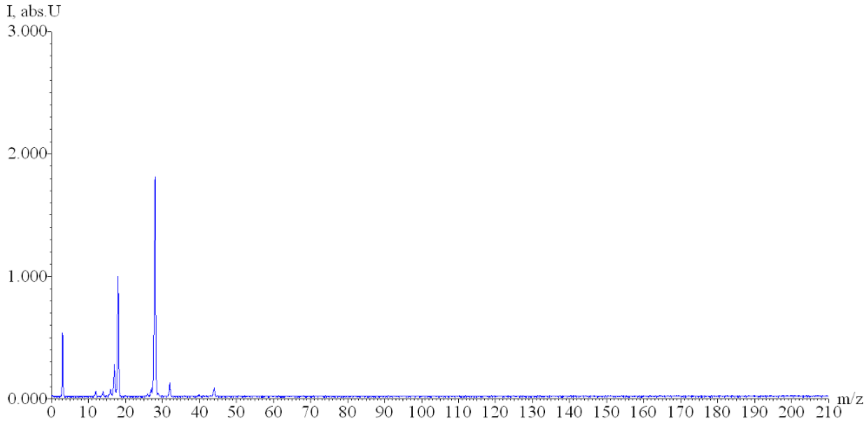
I, abs.U
3.000
2.000
1.000
0.000
0 10 20 30 40 50 60 70 80 90 100 110 120 130 140 150 160 170 180 190 200 210
m/z

(b)

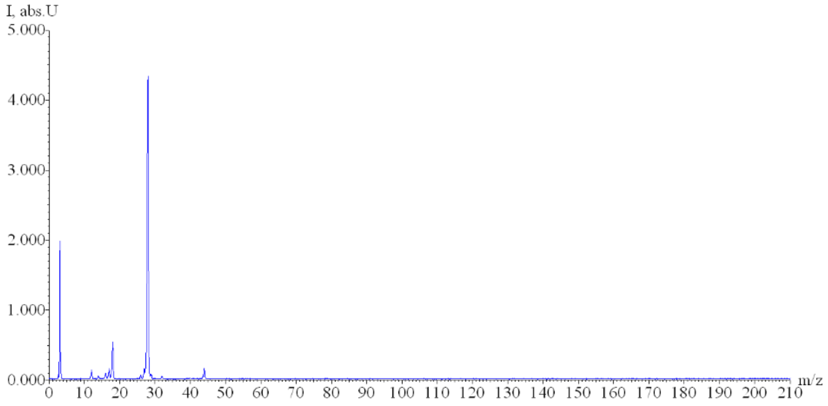
I, abs.U
5.000
4.000
3.000
2.000
1.000
0.000
0 10 20 30 40 50 60 70 80 90 100 110 120 130 140 150 160 170 180 190 200 210
m/z

(c)

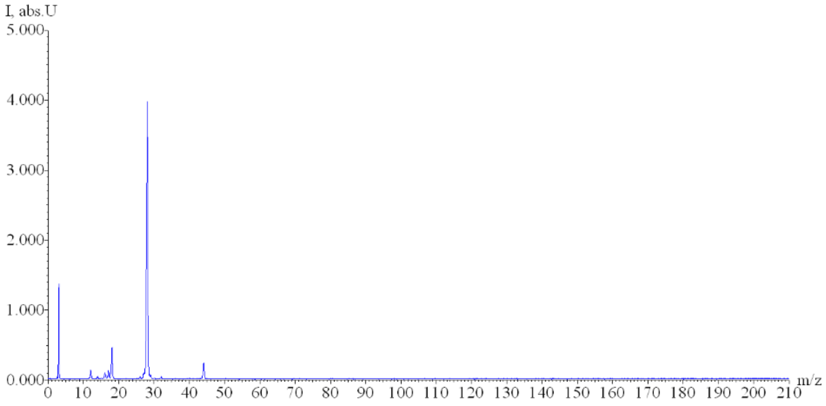
I, abs.U
5.000
4.000
3.000
2.000
1.000
0.000
0 10 20 30 40 50 60 70 80 90 100 110 120 130 140 150 160 170 180 190 200 210
m/z

(d)

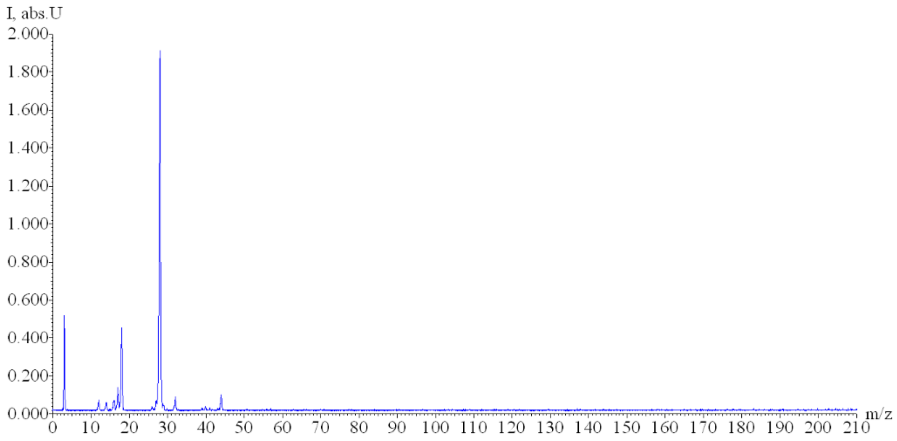

(e)

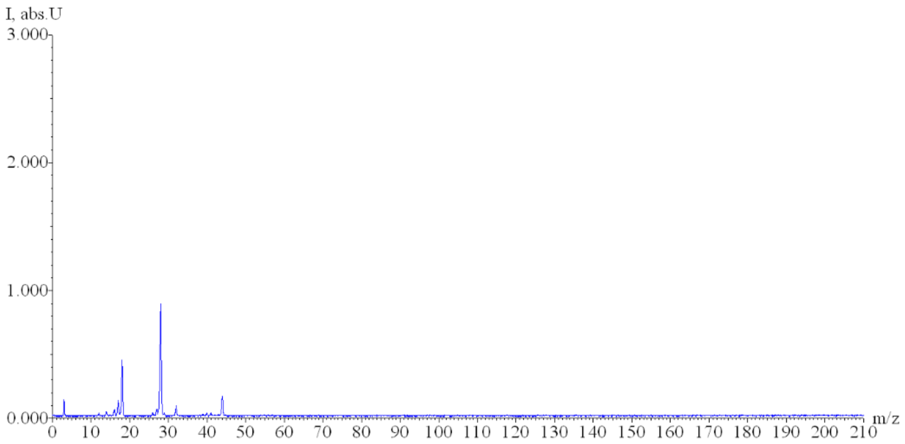

(f)

Figure S1. Mass spectra of the cellulose-based materials pyrolysis products at 280 °C obtained after electron ionization: **(a)** KHC4-600A; **(b)** KHC4-600V; **(c)** KHC4-600VA; **(d)** KHC4-600AV; **(e)** KHC4-600A-1000; **(f)** KHC4-600A-1650.

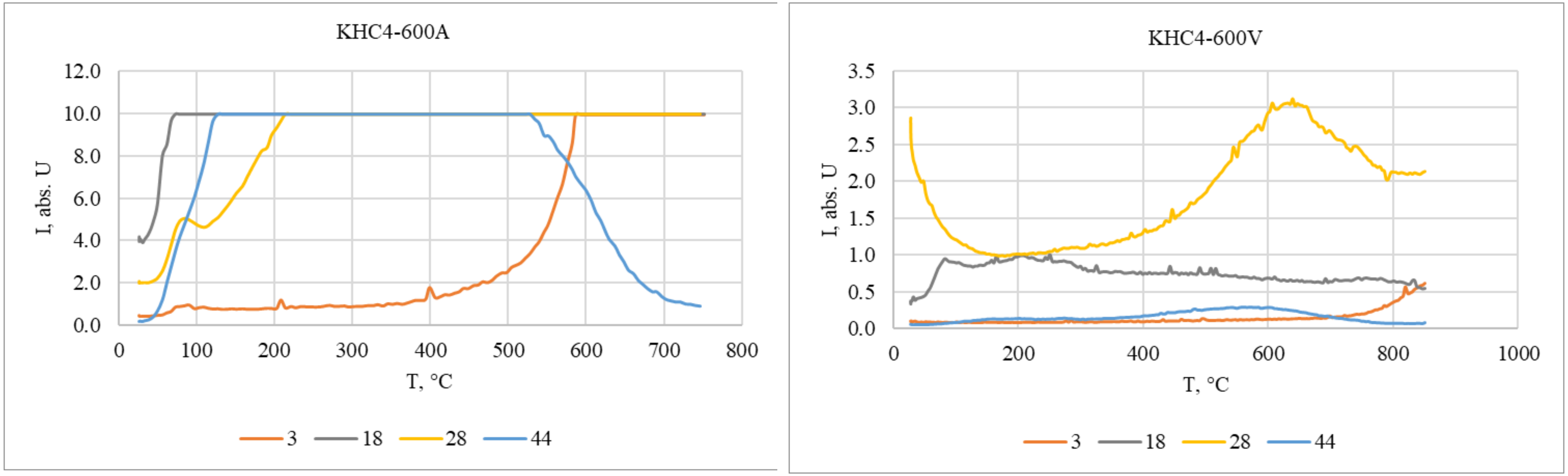
KHC4-600A
I, abs. U
T, °C
0.0
2.0
4.0
6.0
8.0
10.0
12.0
0
100
200
300
400
500
600
700
800
3
18
28
44
KHC4-600V
I, abs. U
T, °C
0.0
0.5
1.0
1.5
2.0
2.5
3.0
3.5
0
200
400
600
800
1000

(a) **(b)**

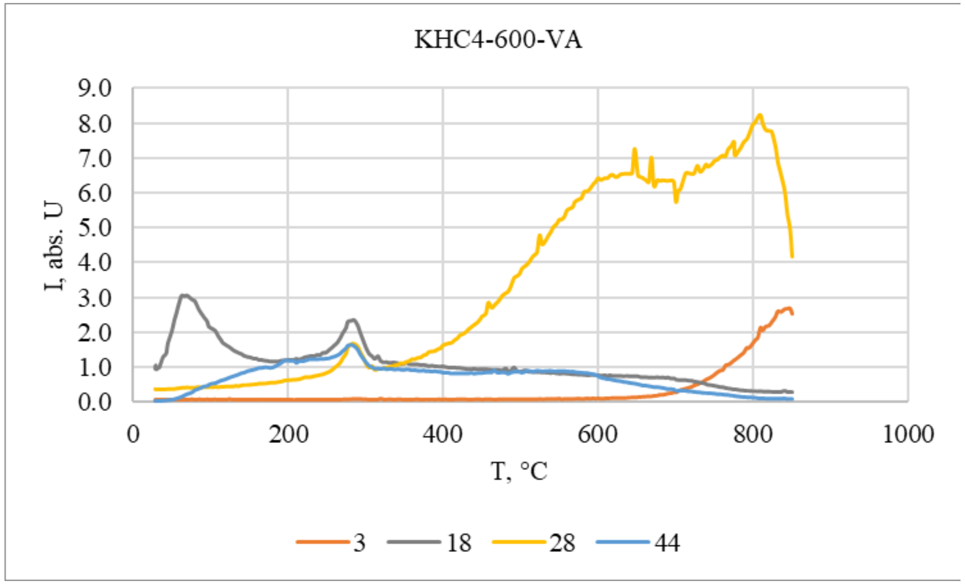
KHC4-600-VA
9.0
8.0
7.0
6.0
5.0
4.0
3.0
2.0
1.0
0.0
I, abs. U
0
200
400
600
800
1000
T, °C
3
18
28
44

(c)

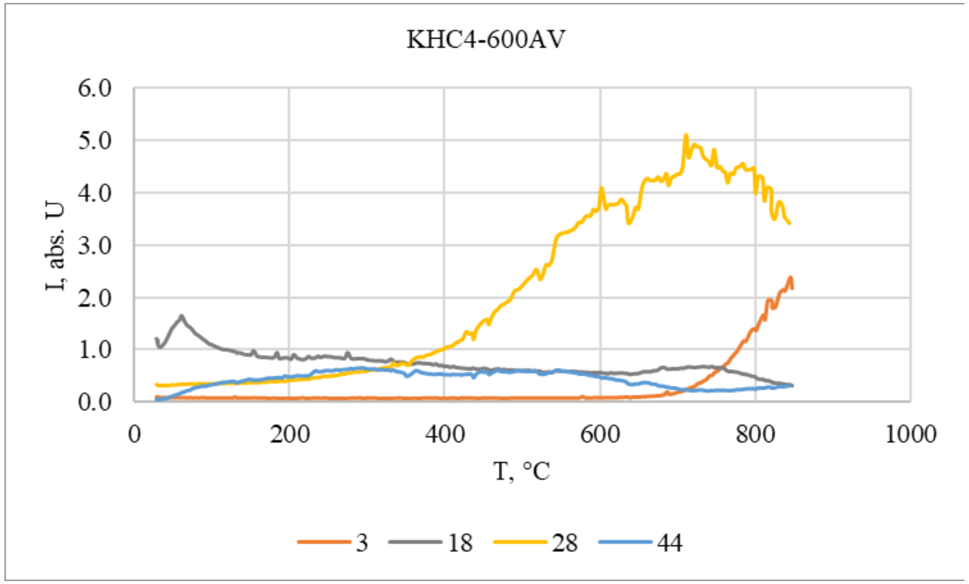
KHC4-600AV
6.0
5.0
4.0
3.0
2.0
1.0
0.0
I, abs. U
0
200
400
600
800
1000
T, °C
3
18
28
44

(d)

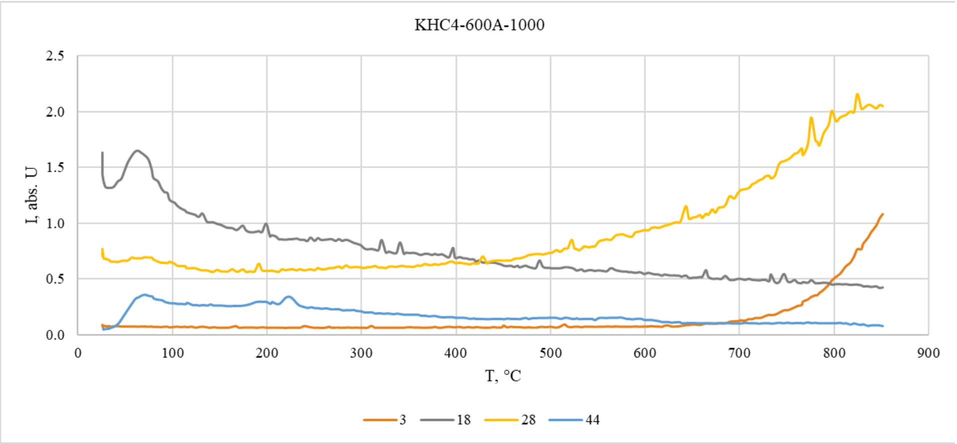

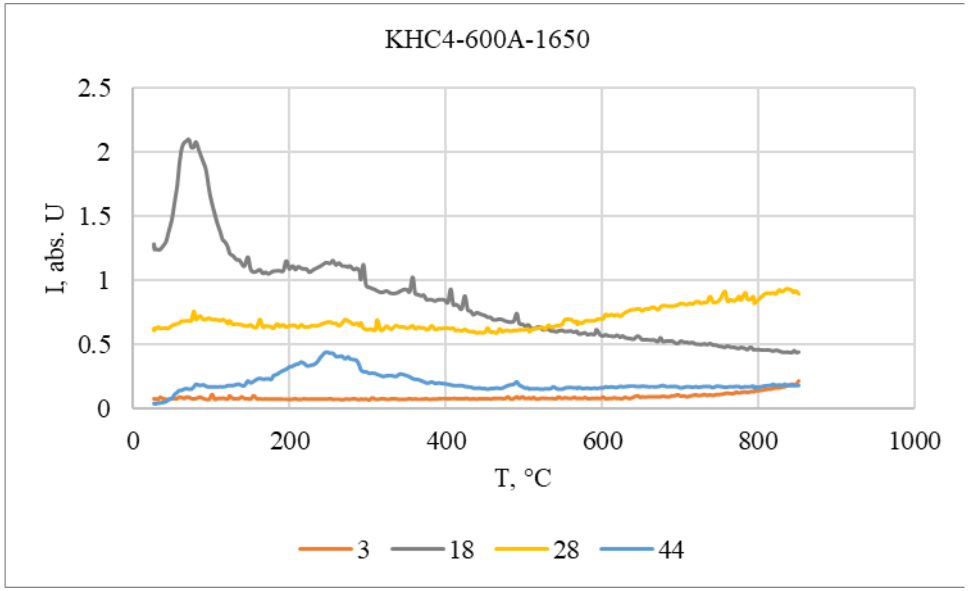

(e) **(f)**

Figure S2: TPD-curves of the ions with *m/z* 3, 18, 28, 44 under pyrolysis of cellulose-based materials: **(a)** KHC4-600A; **(b)** KHC4-600V; **(c)** KHC4-600VA; **(d)** KHC4-600AV; **(e)** KHC4-600A-1000; **(f)** KHC4-600A-1650.
